# Decoding Intention at Sensorimotor Timescales

**DOI:** 10.1371/journal.pone.0085100

**Published:** 2014-02-11

**Authors:** Mathew Salvaris, Patrick Haggard

**Affiliations:** 1 Institute of Cognitive Neuroscience, University College London, London, United Kingdom; University of Turin and the Italian Institute of Technology, Italy

## Abstract

The ability to decode an individual's intentions in real time has long been a ‘holy grail’ of research on human volition. For example, a reliable method could be used to improve scientific study of voluntary action by allowing external probe stimuli to be delivered at different moments during development of intention and action. Several Brain Computer Interface applications have used motor imagery of repetitive actions to achieve this goal. These systems are relatively successful, but only if the intention is sustained over a period of several seconds; much longer than the timescales identified in psychophysiological studies for normal preparation for voluntary action. We have used a combination of sensorimotor rhythms and motor imagery training to decode intentions in a single-trial cued-response paradigm similar to those used in human and non-human primate motor control research. Decoding accuracy of over 0.83 was achieved with twelve participants. With this approach, we could decode intentions to move the left or right hand at sub-second timescales, both for instructed choices instructed by an external stimulus and for free choices generated intentionally by the participant. The implications for volition are considered.

## Introduction

Decoding an individual's intentions is the central aim of brain-computer interfacing (BCI) [1]. Successful BCIs would allow those who lack more efficient modes of communication to interact with their surrounding environment [1], with the promise of major improvements in Quality of Life. In addition, BCIs could provide important scientific insights about the mechanisms of human intentional action. In particular, the ability to decode intentions would strongly enhance the scientific armamentarium used to investigate volition. Current research on volition suffers from an inability to manipulate the input to the voluntary motor system. Most studies have been limited to asking participants to choose, freely but arbitrarily, among a set of equivalent actions, and simply measuring the neural correlates of such ‘internally-generated’ action choice [2].

Decoding intention in real time would open the door to interesting experimental possibilities, such as interventions to facilitate/frustrate intentions, and intention-contigent stimulation. An indication of the scientific interest in intention decoding comes from the mythical status of the ‘Grey Walter experiment’. The philosopher Daniel Dennett recounts [3] Grey Walter's presentation, at an undergraduate medical society in Oxford University, of the following experiment, performed around 1963. Patients with implanted epicortical electrodes viewed a slide show by pressing an ‘advance button’. Grey Walter was able to use intracortical readiness potentials to decode the intention to press the button, and use this as the signal to advance the slideshow. The patients apparently experienced a level of surprise when what they had been about to make happen happened “as if by magic”, but the experiment was never fully published, and a systematic decode-and-intervene study of human intention still remains to be performed half a century later.

Three main neural correlates have been used for decoding intention in the BCI engineering literature. These are steady-state visually evoked potentials (SSVEPs), event-related potentials (ERPs) and sensorimotor rhythms (SMR) [1]. SSVEP-based BCIs rely on flickering visual stimuli that, when foveated, produce EEG oscillations at the stimulation frequency, over the visual cortex. These BCIs achieve high accuracy, but are unsuitable for probing intentions since they rely on the relatively weak attentional modulation of automatic visual processing of an external stimulus. This process is quite unlike normal motor intention, which is often defined by the absence of any external stimulus corresponding to the action. ERP-based BCIs detect specific evoked EEG components. The most successful of these is the P3-based BCI [4]–[6]. The P3 is a late event-related EEG component that is modulated by attention, even in the absence of overt motor responses [7]. P3-based BCIs typically present an array of stimuli, and request participants to attend to the stimulus corresponding to their intention. This induces an increase in P3 amplitude for the corresponding stimulus. Detecting the P3 allows the corresponding action to be performed by an artificial agent.

SMR BCIs generally rely on the oscillatory “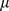-rhythm” modulation observed over the motor cortex during motor execution and imagery. A decrease in the bandpower relative to an appropriate baseline period is referred to as event-related desynchronisation (ERD), while an increase is referred to as event-related synchronisation (ERS) [8]. 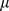-ERD is observed during motor preparation in the contralateral hemisphere to the hand about to move. Motor preparation, motor execution, motor imagery, and even observation of another's actions, all lead to decreases in 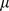-band EEG power [9]–[11]. Other BCIs have been based on event-related desynchronisation in the 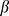 EEG band (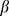-ERD). 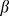-ERD is a pronounced decrease in EEG power around 15–25 Hz over the motor cortex contralateral to the hand that will move or is imagined to move. 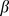-ERS is a rebound in 15–25 Hz power, often observed immediately after motor execution. 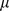-rhythm and 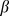-ERD are thought to be robust and intrinsic signals related to intentional motor activation in the brain. SMR BCIs have the lowest accuracy of the three methods described [12]. However, they have the highest ecological validity as paradigms for scientific study of intentional action, because they alone rely on neural correlates closely linked to motor intention. Other ERPs that are often associated with motor intention, CNV, RP and LRP have also been reported. However, the signal-to-noise ratio of these potentials is quite low, and performance of the resulting BCIs has generally been worse than using the sensorimotor rhythms [13], [14].

Therefore, SMR-based BCIs seem particularly appropriate for the scientific study of volition. Most such BCIs rely on detecting changes in sensorimotor EEG oscillations during repetitive real or imagined movements, carried out over a period of several seconds [15], [16]. For example, the BCI user would decide to move their right hand, and would continuously imagine moving the right hand until the BCI detected 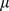-ERD over the left motor cortex, and triggered the corresponding action of the robot, or other artificial agent, at which point the user could cease their motor imagery. The technical details of the BCI effectively transform intention into a continuous feedback-driven effort to control the external device. In contrast, human intentional decisions often seem rapid, changeable, and even phenomenally thin [17]. Thus, to study the processes underlying normal volition, we needed a protocol that would allow us to detect intention to move at the reduced timescales of normal sensorimotor action, and in a single-trial setting.

A standard method for investigating the development of intentions has been the precueing task. In this task, a directional precue is first presented, indicating whether the participant should prepare a movement of their left or right hand. After an appropriate delay, often around 1 s, an imperative cue, or Go signal, instructs the participant to make the action they have prepared. The participant simply prepares the action based on the information gleaned from the direction cue and executes the action at the onset of the imperative/go cue. The degree of preparation, or strength of intention, can be estimated from psychophysiological signals, such as the presence of CNV and 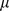-ERD/ERS during the foreperiod between the precue and the Go cue, or by low reaction times to the Go cue. Numerous human and animal studies confirm that intentions to move the left and right hand are developed, and can be changed dynamically by external intervention, during this interval [17], [18].

Most intention decoding studies rely on the model being trained and tested on data collected from participants carrying out the same task. We were interested in whether the model trained during repetitive finger execution/imagination could also be used to decode intention at the sensorimotor timescales of the precueing task. Finally, we wanted to see whether free choices can be decoded as successfully and as early as instructed choices.

A common issue with motor-related BCI is their accuracy and applicability to the general population. The senorimotor 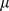 rhythm during real and imagined motor execution is highly variable, both within and between individuals [9], [19]. Therefore, our study of intention decoding, like other studies [20] used only participants who showed modulation of the 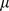 and/or 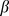 frequencies during the training task. We discuss exclusions and the limitations that they imply later.

## Materials and Methods

### Subjects

Twenty healthy volunteers (six male) participated in the study, all were right handed and between the ages of 19 to 30 (mean age 23). The protocol was approved by the UCL ethics committee, and all subjects gave their written informed consent for the study. All participants first performed a simple repetitive motor execution task (finger tapping). The purpose of this task was to select participants suitable for the BCI experiments. Five participants were excluded at this stage because of EOG and EMG artifacts. Three further participants failed to show any modulation of the 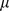/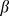 rhythms over the motor cortex related to action execution, so could not be used for motor based BCI. The remaining twelve all showed some modulation of 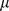 and/or 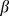 rhythms during execution of the right and/or left finger movement, and were included in the main BCI experiment.

### Experimental protocol

Subjects were seated in a chair at a distance of 80 cm from an LCD screen. Both hands were resting on a keyboard. They were told to remain relaxed, and try to minimise movement and eye blinks. When required to respond, they were told to simply use their index fingers and try to avoid tensing their arms or shoulders. The whole experiment consisted of two model-training sessions and a final testing session. Each session lasted 30–40 minutes with 10–15 minute breaks between sessions. The duration of the whole experiment, including setup, was kept below 3 hours to minimise fatigue.

#### Session 1

The first model-training session was designed to identify EEG signals related to motor execution. Participants were instructed to tap with their right or left index finger at a rate of 2 Hz. They were encouraged to minimise all other movement and to only use the designated index fingers. 50–75 trials were collected for each hand.

#### Session 2

The second session aimed to show that a decoding model based on actual motor execution, derived from the first session, could be used to decode EEG activity in the absence of execution. Participants were instructed to carry out motor imagery of the repetitive finger movement instructed in session 1. All other aspects of the task were as in session 1. This session also allowed us to screen participants for presence of motor-related EEG oscillations, and at least minimal voluntary control over these oscillations.

#### Session 3

In the final session, participants performed a rapid precued, delayed-response task, based on similar tasks in the human motor control literature. Participants were simply instructed to prepare the action indicated by the precue, and to respond as quickly as possible to the imperative stimulus when it appeared. The model trained with the data from the first two sessions could be used to make sample-by-sample predictions of the participant's choice in the delayed response task. The motor task carried out by the subjects, was not the same across the three sessions, but we assumed a common psychological element with a neural correlate present in all tasks. Thus, our BCI decoding would be based on some internal process common to execution (session 1), imagery (session 2), and preparation (session 3) of left or right responses. The existence of such a common element of motor representation is well-established [21], and can be clearly linked to the activity of contralteral motor cortical regions, notably the premotor and motor cortices. We therefore had physiological evidence that all sessions should involve a common process of modulation of motor cortical EEG oscillations.

### Visual protocols

**Figure 1:**
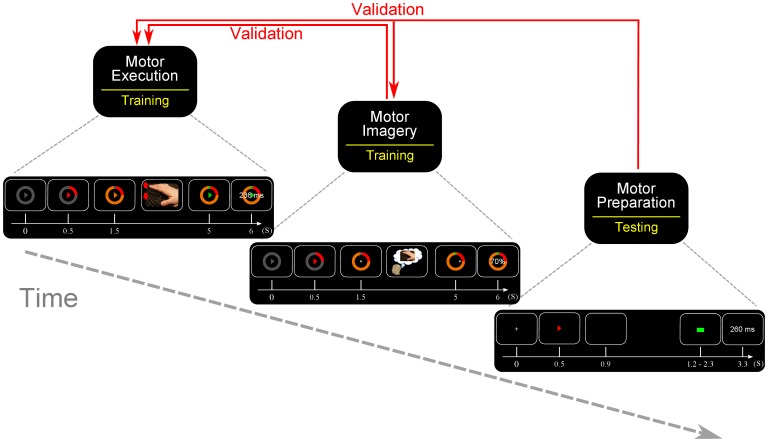
The three protocols used. (far left) Participant carried out real repetitive movements. (centre) Participant carried out repetitive motor imagery and received online feedback. (far right) Simple precueing task where the participant simply reacted as quickly as possible.

The first calibration session can be seen in figure 1. An annulus subtending 2.25 degrees of visual angle appeared at the centre of the screen. After a brief interval, an arrow appeared at the centre of the screen instructing the participant which index finger to use on that trial. The annulus turned progressively red along a clockwise manner, rotating at 90 degrees/s for 500 ms, at which point it turned orange, indicating that participants should begin repetitive finger movement (but without depressing the response key) at a rate of around 2 Hz. After a further 3.5 seconds the annulus and central arrow turned green. The participant was required to respond within 1 s using the same finger they were tapping during the intervening orange phase. The data during the orange phase was used to train the initial BCI model, which could be used for decoding motor imagery during the second session.

The visual protocol for the second session was very similar. After the initial preparation phase the arrow at the centre of the screen changed to a crosshair. The position of the crosshair was updated based on the model output. The position of the crosshair was updated every 17 ms although the model output was calculated every 8 ms. The participant was instructed to use continuous motor imagery to imagine repetitively tapping their left or right index finger (as they had actually done in session 1), according to the arrow direction on that trial. They were instructed not to move their fingers, and this was monitored visually by the experimenter. The participants were rewarded for maintaining the crosshair on or beyond the desired edge of the annulus. For every 8 ms that the crosshair was placed beyond the desired border the participant was rewarded with 0.00014, so if the participant managed to maintain the crosshair beyond the desired border then they would earn an extra 0.062 in that trial. Once the annulus turned green they again pressed the response key, within 1 s.

The final protocol (see figure 1) involved a simple precued delayed response task. Participants were initially shown a fixation cross. After 500 ms, an arrow appeared at the centre of the screen for 400 ms, and then disappeared. The arrow could point either right or left. After a delay period, a Go cue (green square) appeared. Crucially, the interval between directional precue offset and go cue was unpredictably jittered to be between 0.3 and 1.4 s. This jitter encouraged the participants to immediately prepare the designated action, and to maintain this preaparation throughout the delay period, since the Go signal could occur at any time. Participants were instructed to react as quickly as possible to the Go signal. Participants were also rewarded (£1) if their mean reaction time, based on a block of 40 trials, decreased relative to the preceding block of trials. Six participants also carried out a free-choice condition.

In the free-choice condition, they were asked to decide for themselves whether to use the hand instructed by the arrow, or to ignore the instruction and use the other hand. They were asked to make this choice anew when the arrow appeared at the start of each trial, and to try to roughly balance follow and ignore decisions across the block. This protocol was based on paradigms involving voluntary saccade countermanding. They were again rewarded for fast reaction times. In total, for the delayed response task, the participants carried out at least 240 trials.

### Data acquisition

EEG was measured from 27 electrodes according to the international 10-10 system, mounted in a cap. The electrode montage aimed to maximally cover the motor cortices in each cerebral hemisphere, and was based on a grid extending anterioposteriorly and laterally from FT7 to TP8. Horizontal and vertical electroculogram (EOG) was measured with bipolar recording. EMG was recorded from bipolar electrodes placed in a belly-tendon montage over the first dorsal interosseus muscle of both hands. For nine of the twelve participants EMG was only recorded on subjects during the precueing task. For the remaining three EMG was recorded during the whole experiment. Signals from all channels were amplified (g.tec GmgH, Schiedlberg, Austria), filtered (0.1–40 Hz), and digitized (sampling frequency, 128 Hz). The PC carrying out the acquisition also carried out the preprocessing, and during the feedback session, the execution of the BCI model. The visual stimulus display was controlled by a second computer, to ensure accurate timing. Acquisition and model execution were carried out in the Simulink environment. The visual paradigm was written in Python using OpenGl.

### Training the model

The methods used were based on those used for standard motor imagery BCI paradigms [22]. The methods rely on spatial filtering, choosing the optimal time window and frequency bands, and finally training a linear model.

#### Spatial filtering

Spatial filtering is an important part of detecting neuromodulatory changes over the motor cortex [23]. The most widely used and successful method of discriminating such changes has been Common Spatial Patterns (CSP). The CSP approach finds a spatial weighting of the sensors (“spatial filters”) that maximize the variance of the signal in specific EEG bands in one condition while minimizing the variance of the signal of another condition [24]. The variance of a bandpassed signal is equivalent to the band power. Therefore, CSP filters can be used to discriminate conditions that are characterised by high power (ERS) for left hand responses and low power (ERD) for right hand responses, from conditions with the reverse pattern. They are thus effective in decoding intentions to move the left or right hand.

#### Temporal Window selection

A 3.5 second epoch was extracted from the repetitive real and imaginary finger movement conditions used for training. It is assumed that motor execution and motor imagery rely on a common set of mechanisms which underly motor cognition [25]. Furthermore, in [15] it was reported that a model trained on real movement was successfully used to provide feedback during imagined movement. Throughout the literature there is an accumulation of evidence that motor imagery and motor execution share many common features. A subsection of this epoch was determined manually in order to maximise decoding performance for each subject. The period was on average a second long (SD = 0.2 s) and started at least 500 ms after the initiation of the repetitive real/imagined movement. We assumed that this optimal period corresponded to the strongest motor related neural correlates, and that it would provide us with the optimal features for our precueing task.

#### Spectral filtering

The two EEG bands usually associated with motor execution are the 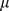 (8–14) and 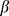 (15–25). The optimal frequency range for decoding was determined for each participant's training data [23].

#### Linear model

Simple LDA (Linear Discriminant Analysis) was used, which has been used in the past to good effect [13], [22], [26]. An LDA is a linear classifier which attempts to find a linear combination of features that separate two classes. So in our tasks it seeks to find a weighting of the log bandpower from the defined spatial sources that would allow it to classify the neural activity as belonging to a right or left hand button press.

The procedure implemented to train the model is similar to the one outlined in [23]. The data from the actual movement and motor imagery sessions was combined. First, the optimal frequencies are determined then, using the filtered data, the optimal time window is found. Each electrode is spatially filtered using a surface Laplacian, then average plots of the spectra in each condition are calculated from the onset of the orange ‘prepare’ cue, to the onset of the go cue. The classwise spectral plots for each channel, as well as their respective squared point biserial correlation coefficients, are used to choose the optimal spectral windows. Then, using the filtered data, the average classwise bandpower plots are used to find the optimal time window. From the filtered data, 27 spatial filters are extracted. From these spatial filters the best four are selected based on eigenvalue magnitude and biological plausibility (e.g., involvement of cortex contralateral to the movement). The EEG data from the 27 channels is spatially filtered into the 4 signals corresponding to the 4 retained filters. The log-variance is taken from each trial of the training data. The resulting feature vector is the same size as the number of spatial filters used. A simple linear classifier is then trained on the calibration data.

### Classifying the delayed response data

When decoding intentions in real-time, it is important to balance accuracy of decoding against speed of intention detection. A conservative decoding approach will require considerable evidence before predicting the intention. Conservative decoding will therefore tend to be more accurate, but detect intention onsets later. In our precued delayed response task, the conservative approach would therefore decode intentions accurately, but closer to the time of actual movement exeuction. This trade-off is apparent when choosing the width of the EEG window used for decoding intentions. Using larger windows would be more accurate but slow, whereas smaller windows would react quicker, allowing us to identify the first onset of direction intention rapidly, but at the expense of accuracy. We therefore compared three different window widths: 500 ms 300 ms and 100 ms. The data was spectrally and spatially filtered using the parameters determined from each participant's training data. The log-variance of the data was then passed to the linear model, which produced a continuous score. The output of the model was thus a continuous value along a dimension, whose extremes corresponded to the left and right index finger movements learned during training. The models ability to classify the data was assessed using the area under the Receiver Operating Characteristic curve (ROC AUC). This is equivalent to a d′ sensitivity measure for detecting the intention to move one hand rather than the other [27].

## Results

### Decoding instructed choice (Instructed)

**Figure 2:**
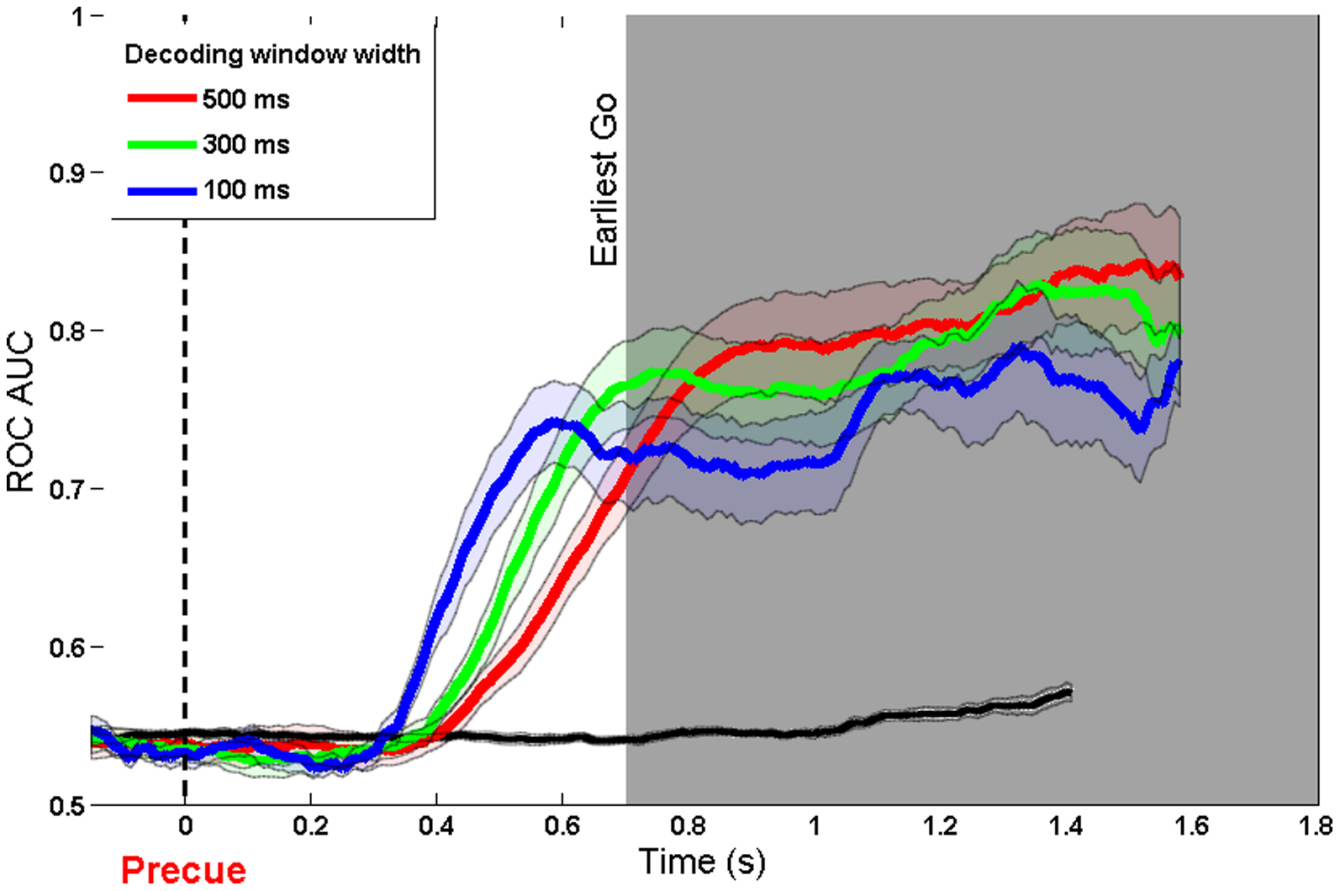
ROC AUC of model trained on real and imaginary movement data and tested on precue data. Aligned to direction cue and using three different window widths (500 ms, 300 ms and 100 ms). The black line shows the decoding accuracy achieved when condition labels were randomly reshuffled.

The model was trained on each participant's real and imaginary movement data, then tested on the precued delayed-response task. The model could decode intentions with good accuracy (AUC
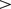
0.83) during the interval between an instruction precue and a Go stimulus of the precueing task. The results are shown in figure 2, time-locked to the direction cue. Several points arise from this figure. First, decoding accuracy begins to rise with 200–400 ms of the directional precue. The short initial delay presumably reflects the time required to visually encode the directional arrow. Second, decoding accuracy rises earlier for shorter decoding windows, as might be expected. In our task, the earliest possible timing of the Go signal was 700 ms after the onset of the directional precue. By this time, decoding accuracy is already high. Data beyond this point is based on progressively smaller number of trials, as the distribution of delay periods was uniform random between 700 and 1800 ms. Decoding accuracy continues to improve during the remaining delay period. This could reflect an increase in preparation: as each moment passes without a Go signal, the participant can be more confident that a Go signal will shortly occur, justifying a greater effort towards preparation.

**Figure 3:**
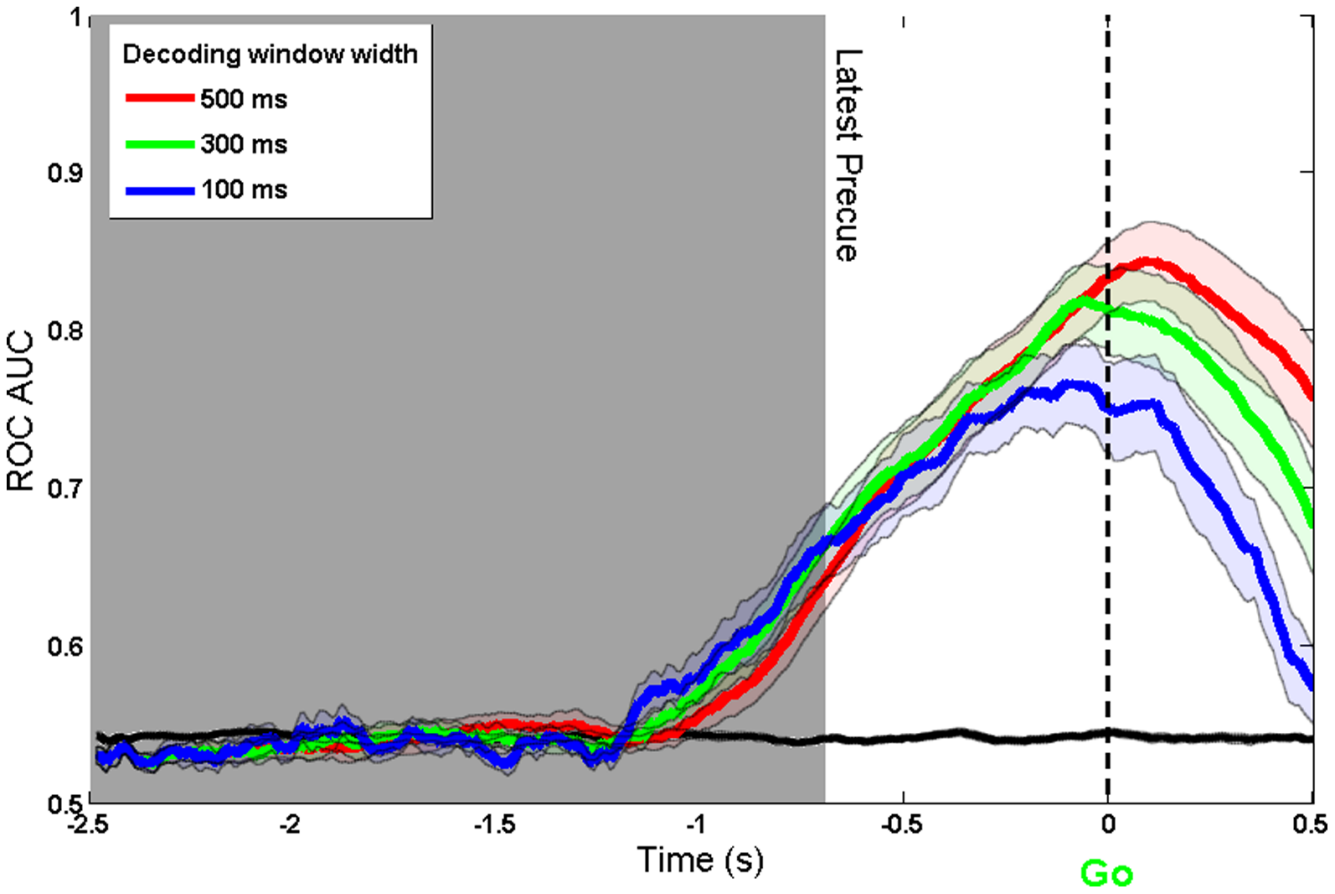
ROC AUC of model trained on real and imaginary movement data and tested on precue data. Aligned to Go cue and using three different window widths (500 ms, 300 ms and 100 ms). The black line shows the decoding accuracy achieved when condition labels were randomly reshuffled.

The data are replotted aligned to the Go cue in figure 3. This presentation is similar to a response-locked average such as a Readiness Potential. The vertical dashed line indicates the onset of the Go cue with the right edge of the shaded aread indicating the latest possible timing of the directional precue. Decoding accuracy increases monotonically and linearly up until the time of action.

**Figure 4:**
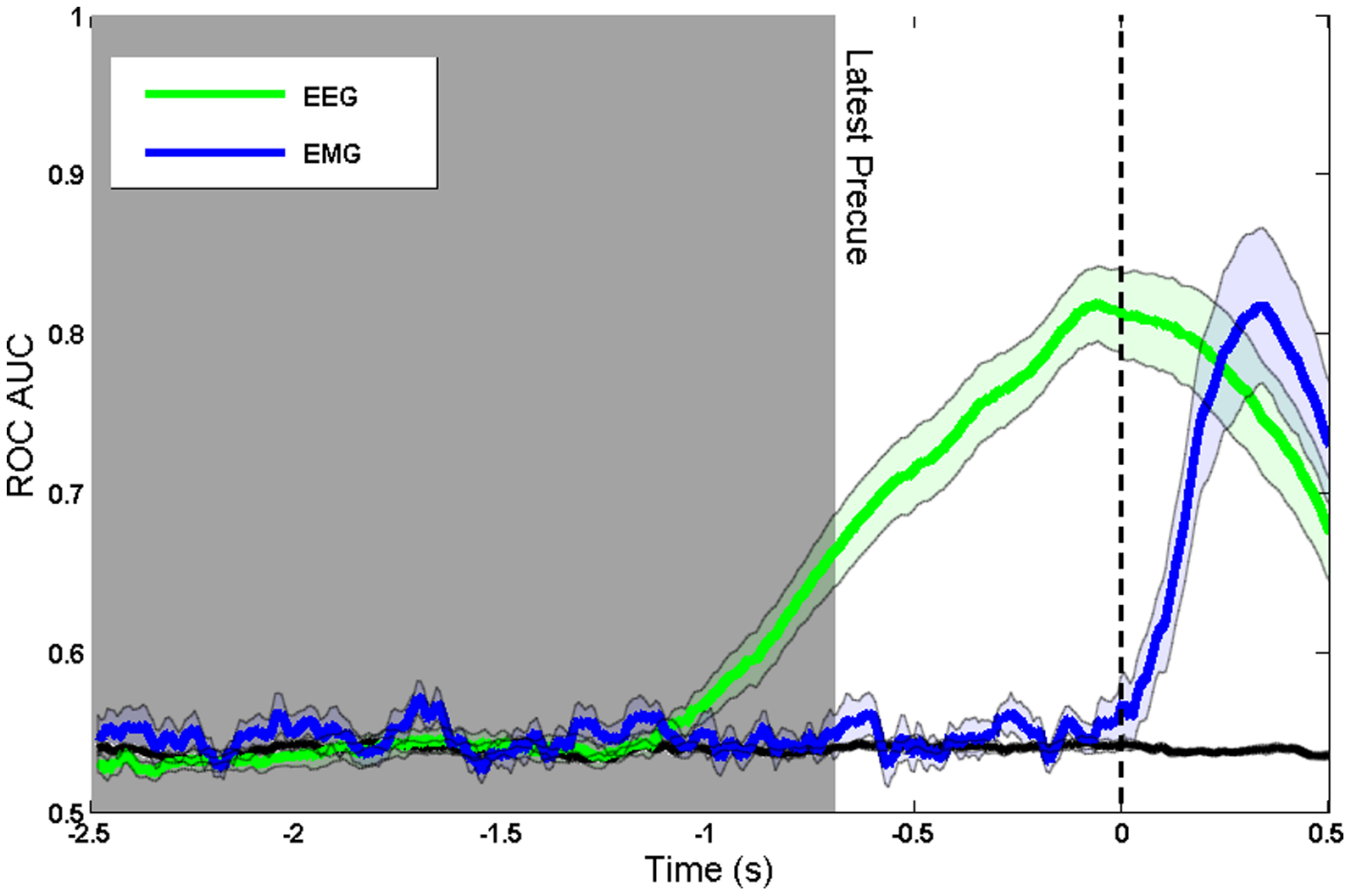
Comparison of decoding using EEG and EMG, when aligned to Go cue. The black line shows the decoding accuracy achieved when condition labels were randomly reshuffled.

To confirm that the decoding was based on brain preparation, and not contaminated by signals related to motor execution, we also analysed the EMG data in the precueing task. We compared the time course of intention decoding using EEG and EMG signals. Figure 4 shows that decoding based on EMG signals becomes possible only after the Go signal, whereas decoding based on EEG is possible during the delay period between precue and Go signal. We conclude that decoding based on sensorimotor rhythms (figures 2 and 3) corresponds to intention and preparation for movement, but not to actual movement itself.

**Figure 5:**
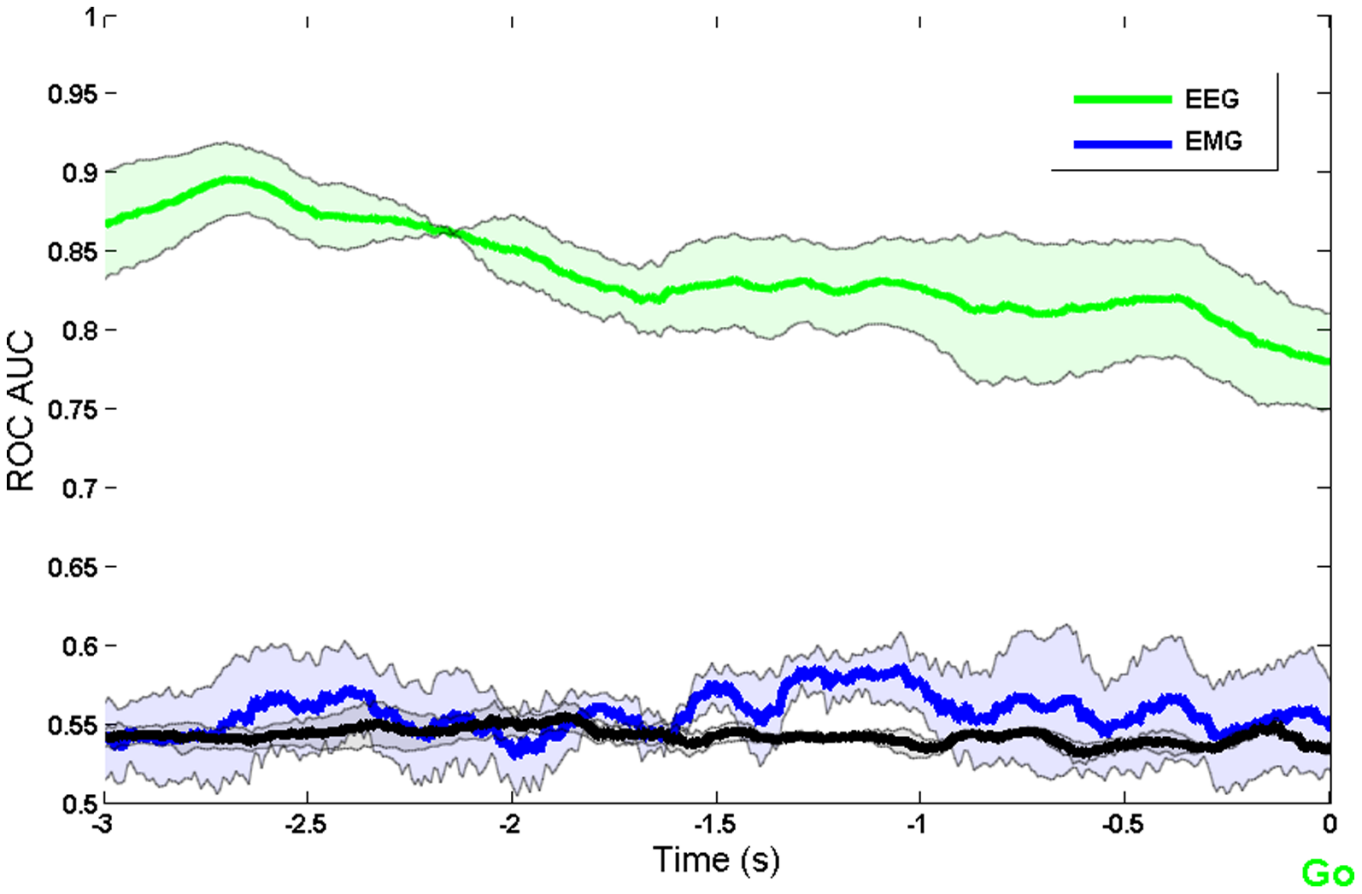
Comparison of decoding using EEG and EMG during imaginary movement task for the last three participants. The black line shows the decoding accuracy achieved when condition labels were randomly reshuffled.

**Figure 6:**
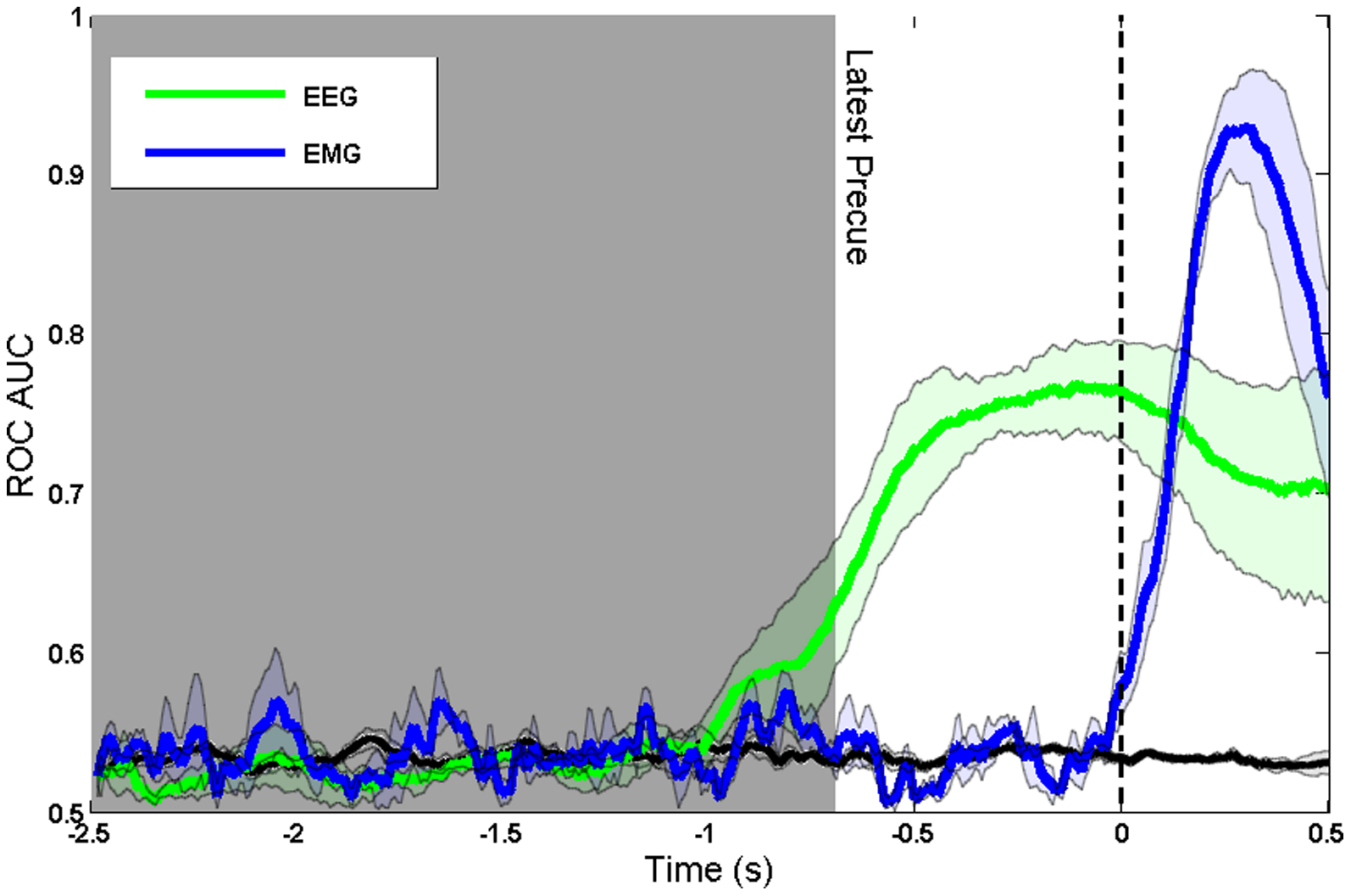
Comparison of decoding using EEG and EMG for the last three participants, when aligned to Go cue. The black line shows the decoding accuracy achieved when condition labels were randomly reshuffled.

For three participants we also recorded EMG during the real and imaginary movement tasks. Figure 5 compares the decoding of movement intention during movement imagery in these participants, based on both EEG and EMG. Figure 6 shows decoding during the foreperiod between precue and Go signal in the precueing task, for the same participants. In both cases, classification achieved using EEG far exceeds that of using EMG. In figure 6, decoding using EEG rises earlier than the decoding using EMG and that at the onset of the Go signal decoding using EMG is very poor. With this we conclude that the model depends on the neuromodulation of the sensorimotor EEG rhythms associated with motor preparation, rather than motor execution.

We next investigated whether a BCI model trained on real and imagined finger movement would provide better decoding during the delay-period of the precueing task, rather than simply using the EEG signals during the delay period, without such prior training. We therefore compared the results using our real and imagined movement training method with a training method confined to the precueing task. For this, we trained the decoding model with one subset of precueing task data, and tested on another subset, using 10 fold crossvalidation. Prior training with execution and imagery gave a modestly but significantly better decoding accuracy (M = 0.83, SD = 0.08) than decoding based only on precueing data (M = 0.76, SD = 0.09): (t(11) = 3.61 p
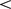
0.005). To investigate the specificity of our CSP approach, we compared our results with an alternative algorithm based on simple surface Laplacian spatial filtering [28], [29]. We found the CSP method (M = 0.83, SD = 0.08) produced modestly but significantly (t(11) = 4.2, p
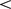
0.002) better results than the surface Laplacian spatial filtering (M = 0.79, SD = 0.08), which is in agreement with other studies [23], [24], [30].

We next investigated whether using a BCI model trained on real and imagined finger movement provides better decoding during the precued delay-period itself than simply using the EEG during the delay period itself. We therefore compared the results using our training method to simply training the model with one subset of precueing task data, and testing on another subset, using 10 fold crossvalidation. Prior training with execution and imagery gave better decoding accuracy (M = 0.83, SD = 0.08) than simply using the precueing data (M = 0.76, SD = 0.09): (t(11) = 3.61 p
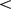
0.005). To investigate the specificity of our CSP approach, we compared our results with an alternative algorithm based on simple surface Laplacian spatial filtering [28], [29]. We found the CSP method (M = 0.83, SD = 0.08) produced significantly (t(11) = 4.2, p
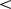
0.002) better results than the surface Laplacian spatial filtering (M = 0.79, SD = 0.08), which is in agreement with other publications [23], [24], [30].

### Free vs Instructed

**Figure 7:**
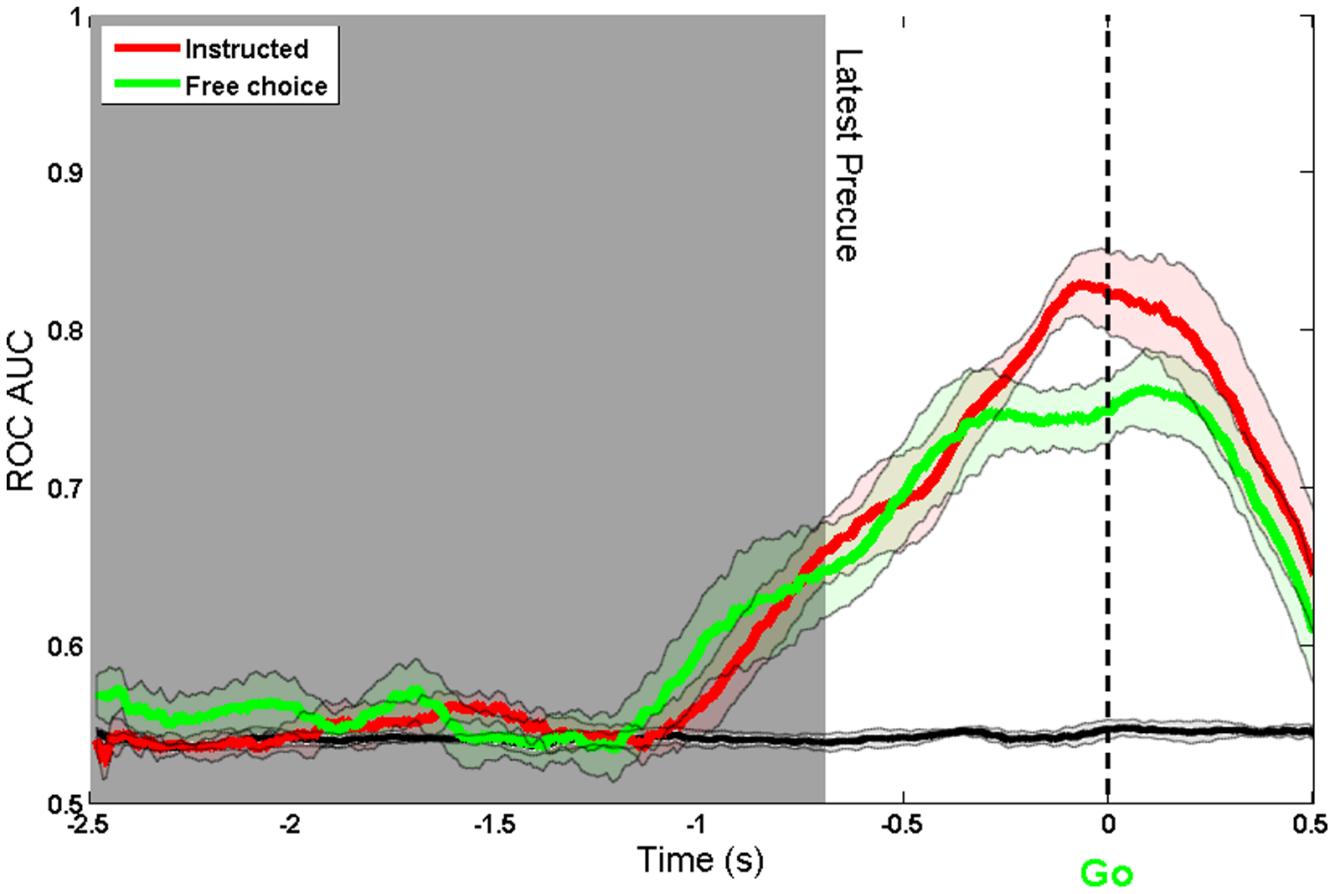
ROC AUC of Free and Instructed choices. Aligned to Go cue using a 300 ms window. The black line shows the decoding accuracy achieved when condition labels were randomly reshuffled.

**Figure 8:**
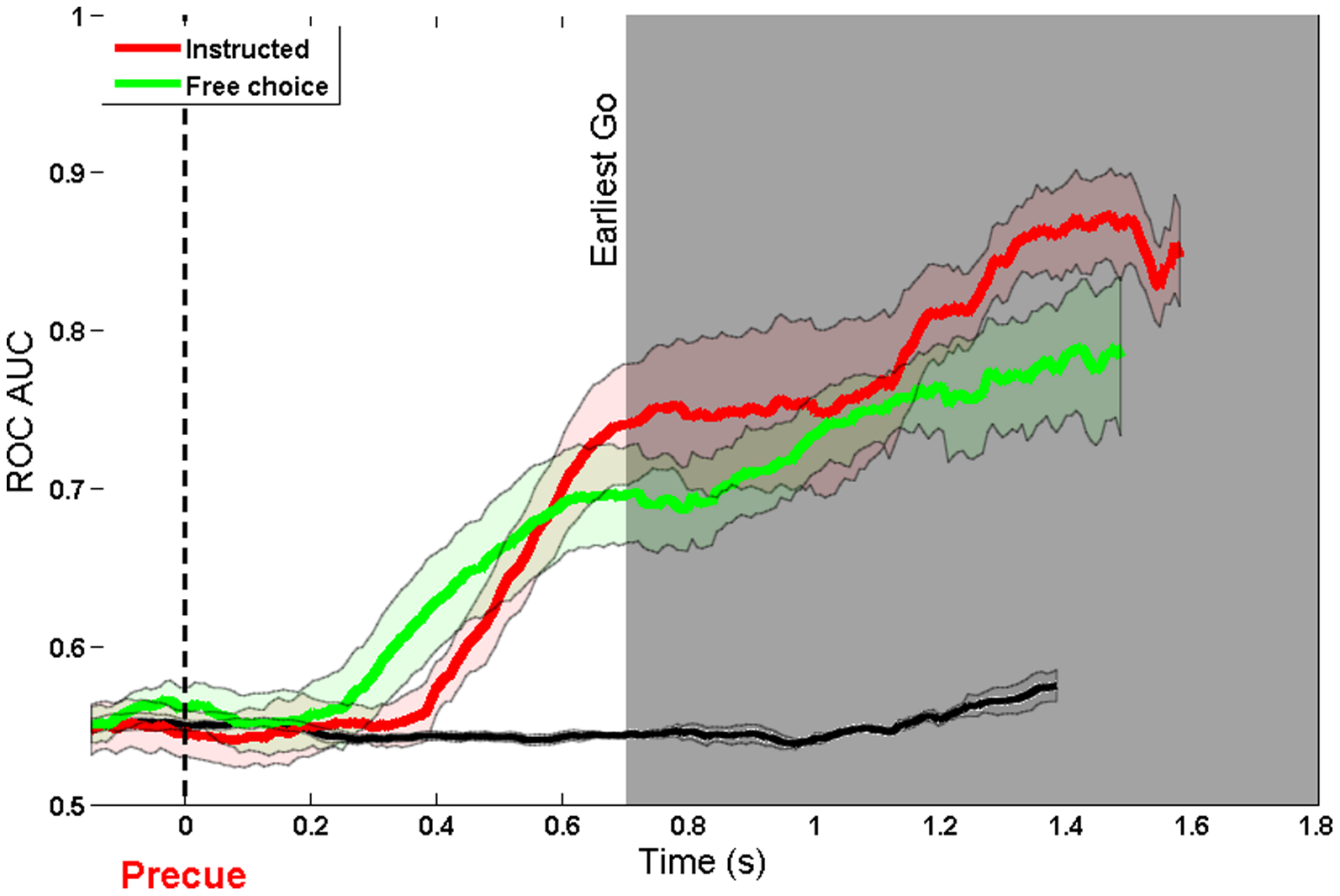
ROC AUC of Free and Instructed choices. Aligned to direction cue using a 300 ms window. The black line shows the decoding accuracy achieved when condition labels were randomly reshuffled.

The ultimate goal of a BCI is to decode internal signals corresponding to an endogenous intention, and not merely to an external instruction. Six of the twelve participants also performed in a condition in which they freely chose which button to press. The directional precue was always shown, and participants were instructed to choose voluntarily either to follow or countermand it. The same model was used to decode the free and instructed decisions. Figures 7 and 8 show decoding accuracy plots for instructed and free choices. The two traces are broadly similar in form, but the free choice data shows some interesting features. First, decoding accuracy begins to rise slightly earlier for free choices compared to instructed choices. To investigate this point statistically, a piecewise linear model was fitted to the free and instructed AUC results of each participant, and the time points of the slope change were compared using a paired t-test. The onset of intention, as indicated by this method, did not differ significantly between conditions (Free (M = 0.43 s after precue, SD = 0.15 s): Instructed (M = 0.52 s, SD = 0.12 s): t(5) = 1.23, p
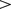
0.27).

Second, decoding accuracy for free choices did not reach as high a level as for instructed choices (see figure 7). We compared decoding accuracy between conditions at the onset of the go cue, since this is logically the key point for decoding sensorimotor intentions. The difference between conditions was significant (instructed (M = 0.82, SD = 0.06): free (M = 0.75, SD = 0.05): t(5) = 2.85, p
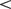
0.04). This difference could potentially reflect simple differences in degree of preparation between conditions, perhaps due to the greater difficulty of free choices. The reaction times to the Go stimulus offer an independent check of how prepared participants were at the time of the Go signal. We found a very small, and non-significant difference between reaction times for free (M = 0.274 s, SD = 0.012 s) and instructed (M = 0.267 s, SD = 0.017 s) conditions (t(5) = 1.37, p
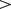
0.22). This suggests that the difference in decoding accuracy does not simply reflect the level of preparation or alertness, but instead corresponds to some more specific factor that differs between free and force choices, such as the differential activation of the two cerebral hemispheres.

**Figure 9:**
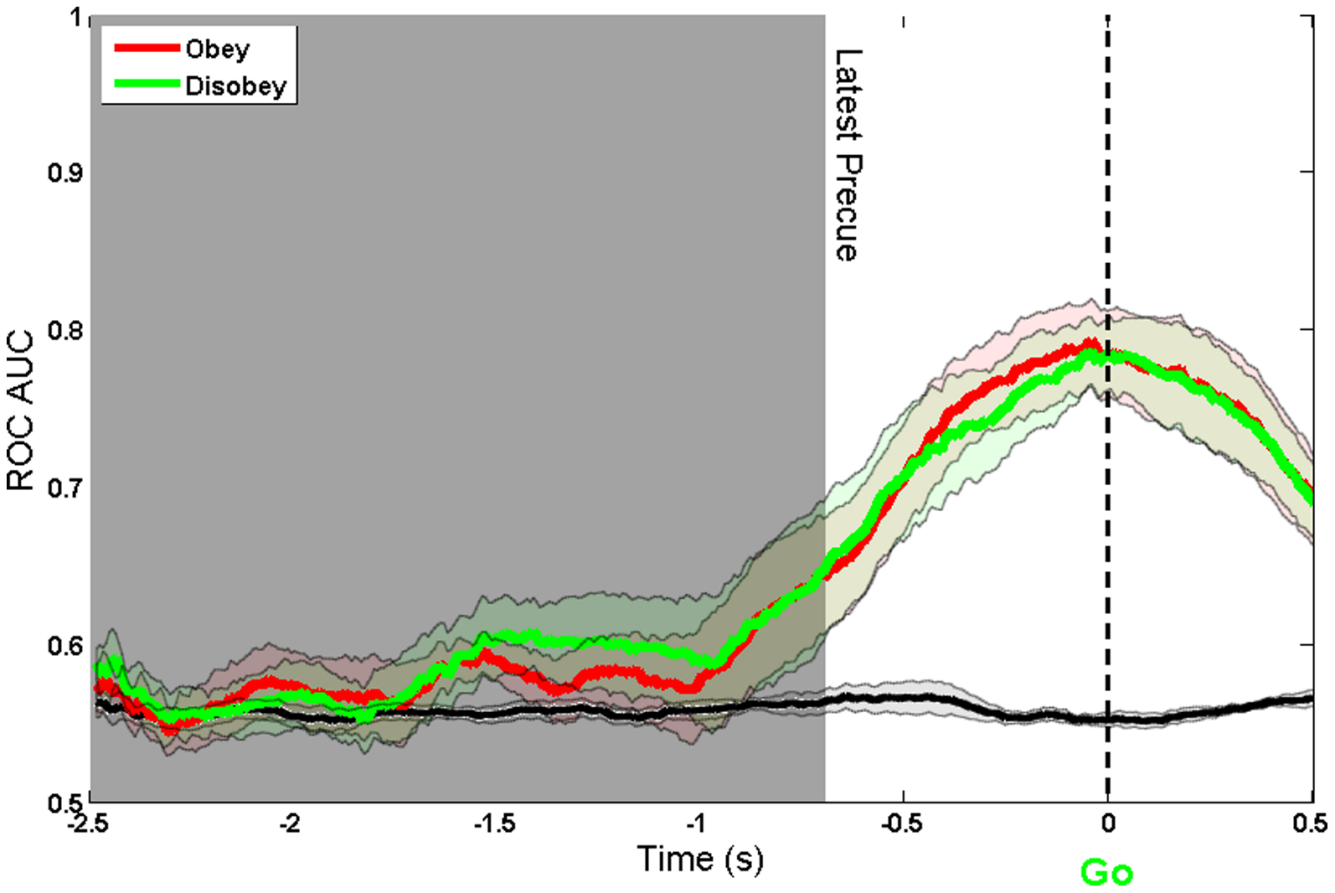
ROC AUC of Obey vs Disobey under the Free condition. Aligned to Go cue using a 300 ms window. The black line shows the decoding accuracy achieved when condition labels were randomly reshuffled.

We also compared decoding for free choice trials where participants decided to obey/follow or disobey/countermand the direction cue. Figure 9 shows that both free-choice outcomes could be decoded equally well. Furthermore, the reaction times for the obey and disobey were almost identical (Obey (M = 0.266 s,SD = 0.025): Disobey (M = 0.267, SD = 0.019): t(5) = −0.07, p
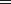
0.95), suggesting that the fact that they obeyed or disobeyed the directional cue had no affect on the amount of preparation or execution of the motor command.

## Discussion

We have decoded human intentions to produce voluntary actions at sensorimotor timescales, using a model trained on repetitive execution and mental imagery. Importantly, the resulting model offers significant benefits in decoding intentions in a precued delayed-response task. In particular, our approach provides a link between the literatures on continuous intention-recognition in BCI applications, and the neuroscientific literature on brain circuits for action preparation. To our knowledge, these two approaches have not been directly integrated before. These results demonstrate that the neural correlates of repetitive executed and imagined movement are also present in the preparation of action. This finding is consistent with the general notion of ‘motor representation’ suggested by neuropsychological theories [21], and points to action preparation as the common element of all three tasks. Importantly, however, the timescales of the three tasks studied here are quite different. We found that models for repetitive execution and imagery were also successful in decoding one-shot intentions in the rapid motor tasks used as laboratory analogues of natural intentional action in the human motor control literature. This result implies that neural codes for a hemisphere-specific action are deployed throughout continuous repetitive action, and that similar neural events occur during sub-second phases of action preparation. We believe this is the first application of BCI-related decoding to sensorimotor timescales of the experimental motor control literature. Clearly, our result does not mean that the neural timescales of both tasks are the same. Rather, our data shows that the neural processes underlying both tasks, notably 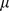 and 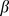 ERD/ERS, have similar spatial localisation and modulation, and that these processes contribute to short-term preparation for intentional action.

Importantly, our method allowed us to decode participant's free decisions regarding whether to make a left or right hand action. The ability to decode volitional decisions is essential for usable BCIs. In addition, accurate decoding of free decisions in real time would allow a valuable methdological step in the scientific study of intentional action. For example, participants in our study could be presented with intention-contingent stimuli. A “STOP” signal could be flashed up just before execution of an ‘undesirable’ action, for example. In previous voluntary action experiments, participants freely chose between using the left or right hand [31]. In our implementation, this corresponded to either obeying/following or disobeying/countermanding the directional precue. This design was based on previous countermanding saccade studies [32], but is formally equivalent to a binary choice between action alternatives. Interestingly, the countermanding approach allowed us to compare the strength of intention for the two possible action choices. One might imagine that intentions are facilitated by following external cues, but are impaired when they are in conflict with an external cue. In fact, we found no discernible difference in strength of intention between choices to obey and disobey. While this null result must be interpreted with caution, particularly from a small study such as ours, we take this as modest evidence that the intentions measured in our study arise at a later processing stage than the stage of conflict resolution. Indeed, conflict resolution for saccades has been linked to the Supplementary Eye Field [32], while our CSP codes were focused on the motor cortical regions contralateral to movement. In brief, our result has the interesting implication that a conflict in the initial choice of action does not impair the subsequent development of intention.

A key finding of our study is that free-choice intentions are less well decoded than instructed intentions. Since we interpret decoding accuracy as a proxy for strength of intention, this finding implies that free-choice decisions lead to weaker intentions than instructed decisions. We found no difference in strength of intention between obey and disobey choices (previous paragraph), we interpret this result as a main effect of volition, rather than a possible effect of conflict. More specifically, we found that free choice intentions began no earlier than instructed intentions, but had only reached a lower level of intention strength by the time of the Go signal. We now discuss the latency and amplitude of free-choice intentions, in turn. Importantly, decoding accuracy at the time of the precue was low in free-choice decisions. This helps to rule out the possibility that participants had merely predecided before the trial which hand they would use, and entirely ignored the precue. Predecision presents a major methodological difficulty in studying voluntary action processes [33]. The methods used in many volition experiments make predecision difficult to rule out. We suggest that strength of intention revealed by decoding is a useful measure to determine when decisions are made [34].

The time-course of decoding suggests that intentions in this paradigm are clearly triggered by the cue, even in the free choice condition. In much of the “free will” literature, the timescales are much longer, and are up to the subject (e.g., Libet et al., 1983; Soon et al., 2008). The a “free” choices made in our paradigm are clearly likely to depend on prestimulus activity somewhere in the brain. However, our method sought to maximise decoding accuracy of these intentions from motor-related activity, and did not involve a whole-brain search for earliest relevant information [35].

The low final level of intention strength for free choices may be problematic for BCI applications, but it is scientifically informative about the processes of volition in the human brain. Why might final strength of intention just before action be lower for free-choice than for instructed actions? We suggest that internally-generated decisions are relatively weakly held compared to externally-instructed decisions. That is, voluntary actions are always subject to “changing one's mind”. This gives voluntary action its unique flexibility and unpredictability, which may confer important evolutionary advantages. The low discriminative strength of free-choice intentions may be linked to the notion that an agent who makes a voluntary choice “could have done otherwise” [36], [37]. The notion that volitional choices are weak and changeable relative to instructed choices has been suggested before [17]. However, previous studies could only probe strength of intention by imposing external ‘switch’ cues, and lacked a naturalistic way to compare intention strength. Our method provides the first direct evidence, to our knowledge, that free-choice intentions differ in strength from instructed intentions.

Although we use BCI methods, our study departs from standard BCI practice in a number of ways, consistent with our scientific objectives. In particular, the arrangement of training and testing data in our study differs from classical BCI. In most BCI settings, the same task is used both to obtain the training data, and to test the model. This crossvalidation approach allows BCI studies to optimise decoding of intentions within that task. In contrast, our method takes an established, plausible BCI paradigm, and demonstrates transfer to a completely different task driven by our scientific interests. Thus, training and testing tasks are completely different. While we show good transfer from the training task to the testing task, it seems likely that the transfer involves some loss of decoding accuracy. Nevertheless, the set of features present during our training is available at sensorimotor time scales and is sensitive to cognitive parameters of natural human action.

We end by acknowledging some limitations of our study. First, the size of our study is relatively small, particularly for the comparison between free and instructed choices. However, it is comparable to other studies in the field [38], [39]. The small sample size means that particular caution is required in interpreting non-significant results. For example, we found that free-choice actions could be decoded slightly, but nonsignificantly, earlier after the precue than instructed choice actions. A larger study might identify this as a significant difference, so we hesitate to make strong interpretations regarding this difference based on the present evidence. Second, our results clearly do not generalise to the population as a whole, as our sample is highly selective. In particular, we excluded several individuals with poor EEG signals, low amplitude sensorimotor rhythms, and poor decoding accuracy. Therefore, our conclusions can only apply to a selected subgroup, and should not be taken as implying the general viability of BCI at sensorimotor timescales. Third, we did not include any strong motivation in our free-choice condition. Participants were simply instructed to choose anew each time the precue appeared, which hand to prepare for subsequent action. In a richer conceptualisation of volition, as in most BCI paradigms, participants should have a reason to make one action rather than another.
